# Bone marrow-derived from the human femoral shaft as a new source of mesenchymal stem/stromal cells: an alternative cell material for banking and clinical transplantation

**DOI:** 10.1186/s13287-020-01697-5

**Published:** 2020-06-30

**Authors:** Katarzyna Drela, Luiza Stanaszek, Konrad Snioch, Zuzanna Kuczynska, Mikolaj Wrobel, Sylwia Sarzynska, Pawel Legosz, Pawel Maldyk, Barbara Lukomska

**Affiliations:** 1 Mossakowski Medical Research Centre, Polish Academy of Sciences, 5, Pawinskiego, 02-106 Warsaw, Poland; 2 Ortopedika, Centre for Specialized Surgery, Warsaw, Poland; 3 Department of Orthopedics and Traumatology, 1st Faculty of Medicine, Medical University of Warsaw, Warsaw, Poland

**Keywords:** Bone marrow, Transplantation, Mesenchymal stem/stromal cells, Total hip arthroplasty

## Abstract

**Background:**

Mesenchymal stem/stromal cells (MSC) are commonly used in regenerative medicine. Among different tissues, iliac crest bone marrow (BM) represents the most exploited source, but its disadvantages are a painful aspiration procedure and low cell number. An alternative, readily available source of MSC for research would be beneficial for regenerative medicine development. This work aimed to propose a new source of bone marrow isolation in which the femoral shaft is taken during total hip arthroplasty (THA).

**Methods:**

In preliminary experiments, three different gradient methods for cell separation (Ficoll-Paque 1.078 g/mL, 17% sucrose gradient, BM seeding fraction) were tested with regard to the time of primary culture, initial cell number, the phenotype, and morphology of MSC. Then human bone marrow MSC derived from two different sources, iliac crest aspirate (BM-MSCi) or femoral shaft (BM-MSCt), were analyzed in terms of cell number and colony-forming ability followed by differentiation potential of MSC into osteo-, chondro-, and adipogenic lineages as well as mRNA expression of a variety of cytokines and growth factors.

**Results:**

Our studies showed that MSC isolated from the bone marrow of two different sources and cultured under appropriate conditions had similar characteristics and comparable propensity to differentiate into mesodermal cells. MSC derived from BM-MSCi or BM-MSCt expressed various growth factors. Interestingly, the expression of EGF, FGF, IGF, and PDGF-A was much higher in BM-MSCt than BM-MSCi.

**Conclusions:**

The results of our study demonstrate that human MSC isolated from the BM of the femoral shaft have similar biological characteristics as MSC derived from the iliac crest, suggesting the femoral shaft as a possible alternative source for mesenchymal stem/stromal cells.

## Background

Mesenchymal stem/stromal cells (MSC) can be isolated from many different tissues. However, until now, bone marrow (BM) represents the most exploited source of MSC in terms of human transplantation, and it has attracted much attention as can be readily obtained in a simple procedure [1, 2]. MSC derived from BM was first described in 1967 by Friedenstein et al. as fibroblast-like colony-forming cells with differentiation potential into osteoblasts, chondrocytes, and adipocytes [3]. Despite the benefits in the content of a large number of hematopoietic stem cells, bone marrow contains a relatively low number of MSC (0.001–0.01%) [4]. Historically, a lot of controversies in the nomenclature of MSC have appeared in the literature concerning their origin and properties especially that basic classification of cells has changed. Based on lack of definition of  proper name for cell type triggers multiple problems, i.e., the same cells with different names; for example, MSCs are defined in multiple ways as mesenchymal stem cells, multipotential stromal cells, and, more recently, medicinal signaling cells [5, 6]. The defined criteria for MSC from 2006 were challenged, and nowadays, the term mesenchymal stem/stromal cells are preferred due to their stromal origin. The acronym MSC concerns a heterogeneous population consisting of multipotential stem cells, committed progenitors, and the other cell types [7]. Bone marrow is a very dynamic and diversified microenvironment, colonized with different cell types including osteoblasts, osteoclasts, adipocytes, macrophages, hematopoietic stem cells, and mesenchymal stem cells. All these cells interact with the soluble factors with autocrine, paracrine, and endocrine activity [8, 9]. MSC in the bone marrow resides in the perivascular region, an environment that controls their proliferation and differentiation [9] [10]. There are still limitations especially in obtaining efficient population of MSC; moreover, the culture methods are not precisely defined [11]. The population number of MSC in bone marrow depends on many factors including patient age, used medicaments, health condition, and bone remodeling diseases [12]. Cell abnormalities have been demonstrated in the bone marrow of iliac crest, following corticosteroid therapy, with a decrease in stem cell population. Recently, it has been shown that steroids initiate adipogenesis and stimulate fat-specific gene expression in bone marrow cells where adipogenic gene expression was promoted [9, 13, 14]. Moreover, it was demonstrated that the number of harvested cells significantly decreased during repeated aspirations, despite of increasing volume of aspirate [15, 16]. Important constraint concerning the aspiration of bone marrow MSCs is a painful harvesting procedure as well as a low number of isolated cells. Commercially available MSCs might be the research alternative; however, high price and low amount of acquired cells are discouraging factors. For that reason, the discovery of alternative sources of MSC could help widen the application of these cells in different areas of regenerative medicine. Due to the discrepancy in published data, it is interesting to investigate whether other BM regions contain multipotent MSC population. We propose a novel source of MSC derived from the bone marrow isolated from the femoral shaft obtained during total hip arthroplasty (THA) surgery.

The aim of our study was a comprehensive analysis of bone marrow-derived cells obtained from two different bone marrow regions: iliac crest and femoral shaft. We established the procedures for isolation and optimized culture conditions of these MSCs. Then, the biological characteristics of MSCs and their functional properties were analyzed. The results of our studies demonstrated that bone marrow MSC derived from the femur (BM-MSCt) are of high quality, characterized by intensive proliferation rate and differentiation potential to mesodermal lineages comparable with iliac crest (BM-MSCi). In terms of different growth factor expressions, the highest mRNA level of EGF, FGF, IGF, and PDGF-A was observed in BM-MSCt. These results indicate that the bone marrow isolated from femoral shaft obtained during hip arthroplasty procedures contained a high number of multipotent MSC with increased regenerative potential.

## Methods

### Bone marrow aspiration from the iliac crest

Bone marrow cells were obtained by iliac crest needle puncture aspiration from 5 healthy volunteers at the age of 25 to 40 years, after receiving written informed consent. Briefly, bone marrow aspirates (5–10 mL) were collected with the syringe with anticoagulant (10 U/mL heparin) and three different methods for cell separation (Ficoll-Paque 1.078 g/mL, 17.5% sucrose gradient, and BM whole fractions seeding method) were tested.

### Bone marrow isolated from the femur

Bone marrow was extracted by the processing of the femoral shaft of 10 patients at the age of 28–65 years, undergoing total hip arthroplasty, following a protocol previously established in our laboratory. To fit and fixate the hip prosthesis stem, the femoral canal is opened and prepared with rasps. While opening, the bone marrow from the femoral canal has been aspirated. The extraction protocol was approved by the ethical review board of the Medical University of Warsaw, and all samples were processed after written informed consent. The exclusion criteria included a history of inflammatory arthritis, metastatic cancer, and disorders affecting bone.

### Commercially available BM-MSC

Commercially available BM-MSC (Rooster Bio) samples were maintained and expanded in vitro in MSC medium (Rooster Bio) in the same manner as freshly isolated BM-MSC cells for comparable results. The culture medium was changed every 3 days. Cells were passaged at 80% cell confluence.

### Isolation of cells from the whole bone marrow sample taken from the iliac crest

According to the original work described by Friedenstein et al. [3], the whole bone marrow samples were processed. After centrifugation, the supernatant containing thrombocytes and erythrocytes was discarded, the cell pellet was suspended in complete MSC growth medium (Lonza, Roster Bio) and seeded on culture dishes.

### Isolation of cells from BM taken from the iliac crest using Ficoll gradient centrifugation

The other method of MSC isolation from bone marrow samples was based on density gradient centrifugation to enrich the mononuclear cell fraction. As a separating medium, Ficoll-Paque (1.078 g/mL) was used according to the previous protocol [17]. Briefly, the diluted bone marrow fraction was carefully overlaid on Ficoll-Paque and then the specimen was centrifuged at 300×*g* for 25 min at room temperature. After density gradient centrifugation, mononuclear cells (MNC) were retrieved from the buffy coat layer by pipetting and washed twice with PBS. The final product was re-suspended in MSC culture medium (Lonza) and seeded at high density (2 × 10^5^/cm^2^) on culture dishes. After removing non-adherent cells, the adherent cells were maintained at standard culture conditions 37 °C, 5% CO_2_. The medium was subsequently changed twice a week.

### Isolation of cells from BM of the iliac crest by 17.5% sucrose gradient centrifugation

The third method of bone marrow cell isolation was based on a 17.5% sucrose solution (Sigma) that was used as a separating medium[17]. The volume of 10 mL bone marrow aspirate was collected from patients’ iliac crest under aseptic conditions. The aspirate was diluted 1:1 in phosphate-buffered saline (PBS) and gently overlaid onto the sucrose gradient using the14 gauge aspiration needle. The tubes were centrifuged at 1500 rpm (200×*g*) for 5 min to obtain the buffy coat layer. The buffy coat (approximately 5–7 mL) containing concentrated BM-MSC was collected from the plasma interface and seeded at a density of 2 × 10^5^ cells/cm^2^ in culture flasks containing growth medium (Lonza) and incubated at 37 °C in a humidified environment with 5% CO_2_.

### Bone marrow cells isolated from the femur

Briefly, 2 mL of bone marrow obtained from the femoral shaft were mixed (1:1) with a PBS buffer containing heparin. Then, the cell suspension was centrifuged at 500×*g* for 10 min, and the pellets were suspended in complete MSC medium and cultured in 25-cm^2^ flasks at 37 °C in a humidified atmosphere containing 5% CO_2_.

### BM-MSC culture

In all isolation protocols, MSC cell suspension was seeded in plastic tissue flasks with commercial MSC medium (Rooster Bio) at an initial plating density of 1 × 10^6^ cells/mL using a direct plating method. Then MSC was isolated based on their ability to adhere to the culture plates. After 48 h, red blood cells and other non-adherent cells were removed and washed with PBS, and then the fresh medium was added to allow further cell growth. The culture was incubated at 37°C in 5% O_2_ until complete confluent monolayer cell culture was reached. The adherent MSC grown to 80% confluency in 4–5 days was defined as passages zero (P0). Cultured cells were expanded by passaging. The culture medium was changed every 3 to 4 days. When the first passage became nearly confluent, the cells were re-cultured in similar conditions. For further experiments in this study, we used bone marrow MSC at passage 3, in a decent growth state.

### Analysis of BM-MSC growth

For comparison of the growth potential of BM-MSC derived from different sources, the number of cells was estimated in each passage up to passage 10 of culture. Briefly, cells were seeded with a density of 3 × 10^3^ cells/cm^2^ and cultured for 3 days at standard culture conditions (37 °C and 5% CO_2_). At the same stage of the culture at approximately 80% confluences of growth, the cells were enzymatically detached by adding trypsin/EDTA and counted in the Bürker chamber with the Trypan blue exclusion method. The number of cells was analyzed by calculating population doubling (PDT) time in culture with the formula PDT = t*ln(2)/ln(Ni/N0).

### Metabolic activity CCK-8 assay

BM-MSC isolated from the different sources being in the culture at passage 3 was used for CCK-8 assay. Briefly, 150 μL of cell suspension at a concentration of 1 × 10^3^cells/mL was seeded in a 96-well plate. At the designated culture time points, the cells were washed twice with PBS. Then 90 μL medium (Rooster Bio) and 10 μL of Cell Counting Kit-8 were added to each well. After 2 h of incubation, the absorbance at 450 nm was measured using a microplate reader (Omega).

### Colony-forming unit assay

Colony-forming unit (CFU) fibroblast assays were performed by seeding BM-MSC at density (10^5^cells/cm^2^) on tissue plates and cultured for 10 days in standard conditions; the medium was changed twice weekly. After 10 days of culture, the medium was removed; cells were fixed and stained with 1% crystal violet. Cell colonies containing ≥ 50 cells were counted and calculated as the fraction of per million MNCs seeded. CFUs were examined in duplicates.

### Senescence analysis

For senescence analysis, BM-MSC was seeded in four or five replicates in 24-well plates at 3 x 10^3^ cells/cm^2^. After 2–3 days, when cell cultures reached approximately 70% confluence, the senescence was evaluated using senescence β-galactosidase staining kits (Senescence Cells Histochemical Staining Kit) following the manufacturer’s instructions. Cells were imaged using a microscope mounted with a digital camera. The number of senescent cells and the total number of cells were counted independently by two investigators. 

### Immunofluorescence and flow cytometry analysis of BM-MSC

Bone marrow cells derived from different sources were characterized with regard to their mesenchymal phenotypes. Flow cytometry data were obtained using a FACSCalibur II (BD Biosciences). The MSC Phenotyping Kit BD Stemflow hMSC Analysis Kit was used according to the manufacturer’s information. To obtain data of expression of CD73 (APC-conjugated), CD90 (FITC-conjugated), CD105 (PerCP-Cy5.5-conjugated) and negative CD14, CD20, CD34, and CD45 (all PerCP-conjugated) markers were used. After 72 h of culture at 37 °C/5% CO2, cells were detached by adding trypsin/EDTA, counted and stained with 10 μL of the MSC Phenotyping Cocktail and the respective Isotype Control Cocktail. At least 30,000 events were measured. The remaining unstained cells were seeded on flasks (1 × 10^4^ cells/cm^2^) to perform the same analysis at a higher passage. The data analysis was performed with the software FACS Diva version 6.1.3. Gates were firstly set based on the granularity and size of the cells of interest with the help of side and forward scatter. Subsequently, cells from this gate were shown in the respective fluorescence channels based on the attached fluorochrome.

### Analysis of multilineage differentiation potential of BM-MSC

Differentiation potential of BM-MSC into three lineages, osteo-, chondro-, and adipogenic, were performed in a specific differentiation media at 2/3 passage according to the manufacturer’s instruction.

#### Osteogenic differentiation of MSC

Induction of BM-MSC into osteogenic lineage was achieved by using the StemPro osteogenesis differentiation kit (Life Technologies). BM-MSC obtained from different sources was cultured in differentiating medium for 21 days with medium change 3 times a week. After 21 days, cells were washed with PBS, fixed with 4% PFA (paraformaldehyde), and stained with 2% alizarin red to expose calcium deposition in differentiated osteocytes.

#### Chondrogenic differentiation of MSC

For chondrogenic induction, micro-mass cell culture was generated by seeding 5 μL of BM-MSC suspension in a multiwell plate. Cells were incubated for 30–120 min in a high humidity atmosphere, after which pre-warmed StemPro chondrogenesis (Life Technologies) differentiating medium was added to the culture and incubated at 37 °C at 5% CO_2_ for at least 2 weeks with the medium change every third day. After 14 days, the chondrogenic cells were processed for Alcian Blue staining according to the manufacturer’s protocol.

#### Adipogenic differentiation of MSC

BM-MSC were seeded 1 × 10^4^/cm^2^ in a 24-well plate and cultured for 3–4 days or until they became 80% confluent. After reaching the confluence, the medium was replaced with StemPro adipogenesis (Life Technologies) differentiating medium and incubated for 3 weeks at 37 °C and 5% CO_2_ with the medium changed twice a week. After incubation, the cultured cells were fixed with 4% paraformaldehyde. The adipogenic staining was performed using Oil Red O stain.

### Gene expression analysis

During routine passaging of culture-expanded BM-MSCs (p2/3), RNA was isolated from cells and used to measure mRNA gene expression of MSC markers and growth factors. The primers used in the study are shown in Table 1. The whole cell lysates were prepared from BM-MSC. Total RNA was extracted using Total RNA kits (Total RNA Mini, A&A Biotechnology) and cDNA was synthesized using High-Capacity cDNA Reverse Transcription kits (Thermo Fisher Scientific) according to the manufacturer’s instruction. Gene expression measurements were performed according to the MIQE guidelines [18]. Quantitative polymerase chain reaction (qPCR) was performed using SyberGreen MasterMix, according to the manufacturer’s protocol. All qPCR experiments were performed in triplicate using Applied Biosystem StepOne Software v2.2.2. The relative mRNA expression was calculated using the ΔΔCt method. All mRNA expression data were normalized to the reference gene-glyceraldehyde-3-phosphate dehydrogenase (GAPDH).

**Table 1:** The list of primers used in the studies

Gene	Product length	5’-3’ sequence			
CD73	241	F	CGCAACAATGGCACAATTAC	R	CTCGACACTTGGTGCAAAGA
CD90	236	F	CTAGTGGACCAGAGCCTTCG	R	TGGAGTGCACACGTGTAGGT
CD105	165	F	CACTAGCCAGGTCTGGAAGG	R	CTGAGGACCAGAAGCACCTC
CD166	217	F	CGCAATGCAACAGGAGACTA	R	GGCTAGATCGAAGCCTGATG
vimentin	170	F	GAGAACTTTGCCGTTGAAGC	R	TCCAGCAGCTTCCTGTAGGT
collagen	332	F	AGTGGTTACTACTGGATTGACC	R	TTGCCAGTCTCCTCATCC
fibronectin	386	F	CTGGGATGCTCCTGCTGT	R	CTGTTTGATCTGGACCTGCAG
nestin	209	F	CAGCTGGCGCACCTCAAGATG	R	AGGGAAGTTGGGCTCAGGACTGG
EGF	94	F	GACTTGGGAGCCTGAGCAGAA	R	CATGCACAAGTGTGACTGGAGGT
FGF	172	F	GGTGAAACCCCGTCTCTACA	R	TCTGTTGCCTAGGCTGGACT
IGF-1	240	F	TCGCATCTCTTCTATCTGGCCCTGT	R	GCAGTACATCTCCAGCCTCCTCAGA
TNF α	252	F	GCTGCACTTTGGAGTGATCG	R	ATGAGGTACAGGCCCTCTGA
TGF β	292	F	GTACCTGAACCCGTGTTGCT	R	TAGTGAACCCGTTGATGTCCA
BDNF	153	F	CAGGGGCATAGACAAAAGC	R	CTTCCCCTTTTAATGGTC
PDGFA	227	F	CCCCTGCCCATTCGGAGGAAGAG	R	TTGGCCACCTTGACGCTGCGGTG
IL-1β	129	F	GGGACAGGATATGGAGCAACA	R	TCTTTCAACACGCAGGACAG
IL-6	81	F	GGTACATCCTCGACGGCATCT	R	GTGCCTCTTTGCTGCTTTCAC
VEGF-A	91	F	ATGACGAGGGCCTGGAGTGTG	R	CCTATGTGCTGGCCTTGGTGAG
GAPDH	108	F	ATGGGGAAGGTGAAGGTCG	R	GGGGTCATTGATGGCAACAATA

### Statistical analysis

The results are presented as mean value ± SD. One-way ANOVA variance analysis was performed with the Bonferroni post hoc. The significance level was *p-*value < 0.05. The analysis was performed in the PS IMAGO program (Predictive Solutions, Poland).

## Results

### Optimization of different isolation procedures for BM-MSC

**Fig. 1:**
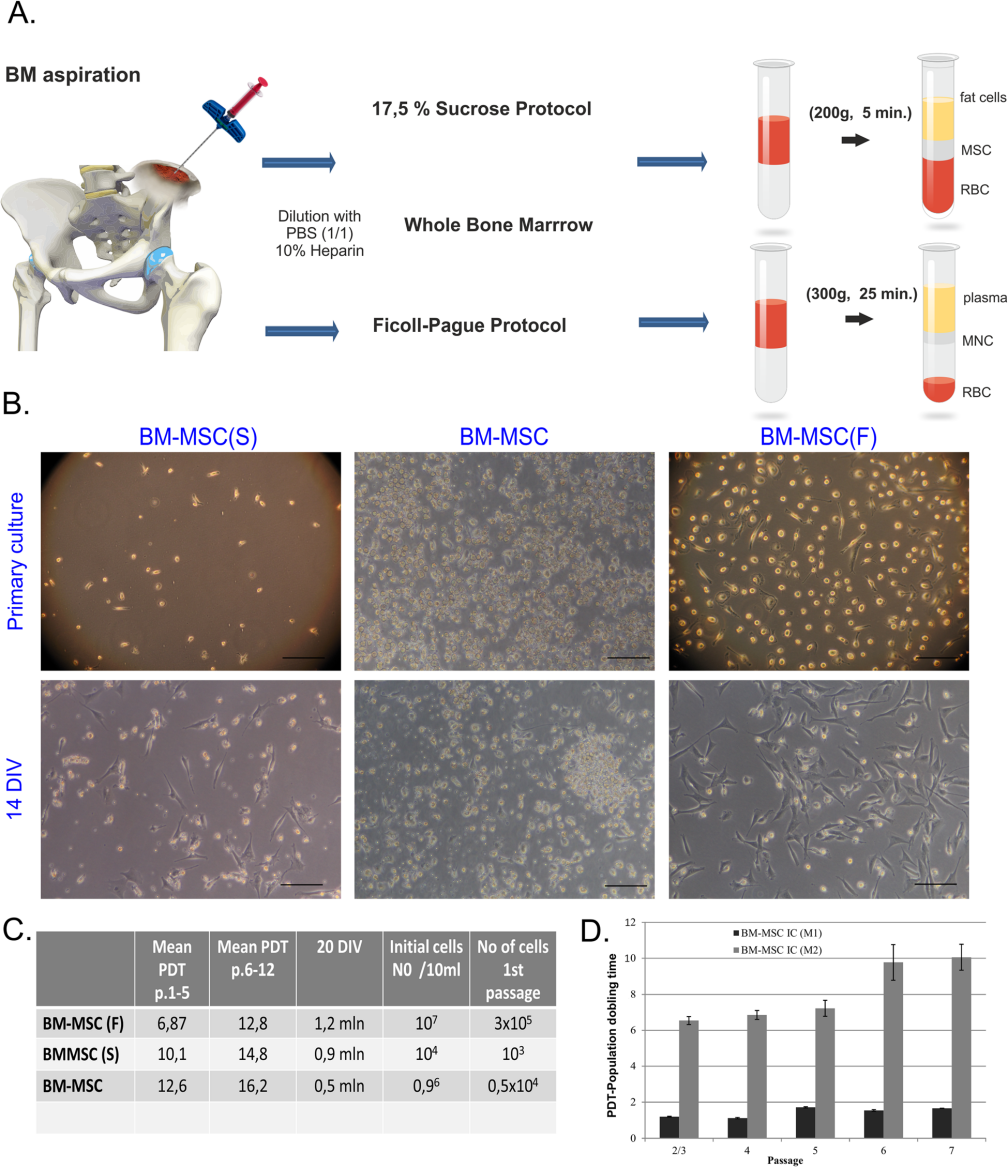
Primary culture of iliac crest BM-MSC and cell morphological characterization at different culture stages—**a** the comparison of three different isolations protocols. **b **Visualization of bone marrow-derived cells cultured at initial passage 0 and after 14 DIV. **c** The number of mononuclear cell concentration obtained after different gradient centrifugations. (F) Ficoll, (S) 17.5% sucrose; the whole fraction of BM and the total cell count for MSC. **d** Graphs show population doubling time (PDT) of BM-MSC in commercial medium 1 versus medium 2. Scale bars = 200 μm. All data reflect mean ± SD from ≥ 5 independent experiments

In order to optimize the cell isolation procedure, we have compared different protocols for iliac crest bone marrow BM-MSCi isolation in terms of their functional activity. We tested three different protocols: (i) Ficoll-Paque gradient, (ii) 17.5% sucrose gradient, and (iii) the explant culture method (Fig. 1a). In all procedures, the bone marrow samples were incubated for 48 h in 37 °C and 5% CO_2_ atmosphere and then washed to remove floating cells. MSCs appeared as plastic-adherent cells within 8–10 days when isolated by gradient methods and approximately 14 days if the explant method was performed. Then, BM-MSC obtained by different isolation procedure was expanded in vitro (Fig. 1b). We calculated the potential cell number after ąround 20 days of culture using two different commercial media (Lonza or Rooster-Bio). According to our results, BM-MSC cultured in the Rooster Bio medium had a better proliferation rate. A clinically relevant dose of cells was achieved after 4 weeks of culture (at the stage of passage 2) (Fig. 1c). Based on the obtained results, we selected the Rooster-Bio culture medium as the optimal for further experiments (Fig. 1d). The phenotype of BM-MSC separated by various techniques did not differ significantly among the three groups. All cells exhibited spindle-shaped morphology, presented typical MSC markers (CD73, CD90, CD105, CD166), and presented multidirectional differentiation potential. However, cell separation using a Ficoll-Paque gradient method enabled us to obtain the higher number of cells, shorter primary culture time, and increased proliferation rates compared with the other two techniques. To conclude, Ficoll-Paque gradient method seems to be the best choice for BM-MSC isolation in order to use them in clinical practice. For that reason, in further experiments, we applied the Ficoll-Pague protocol for BM-MSC isolation.

**Fig. 2:**
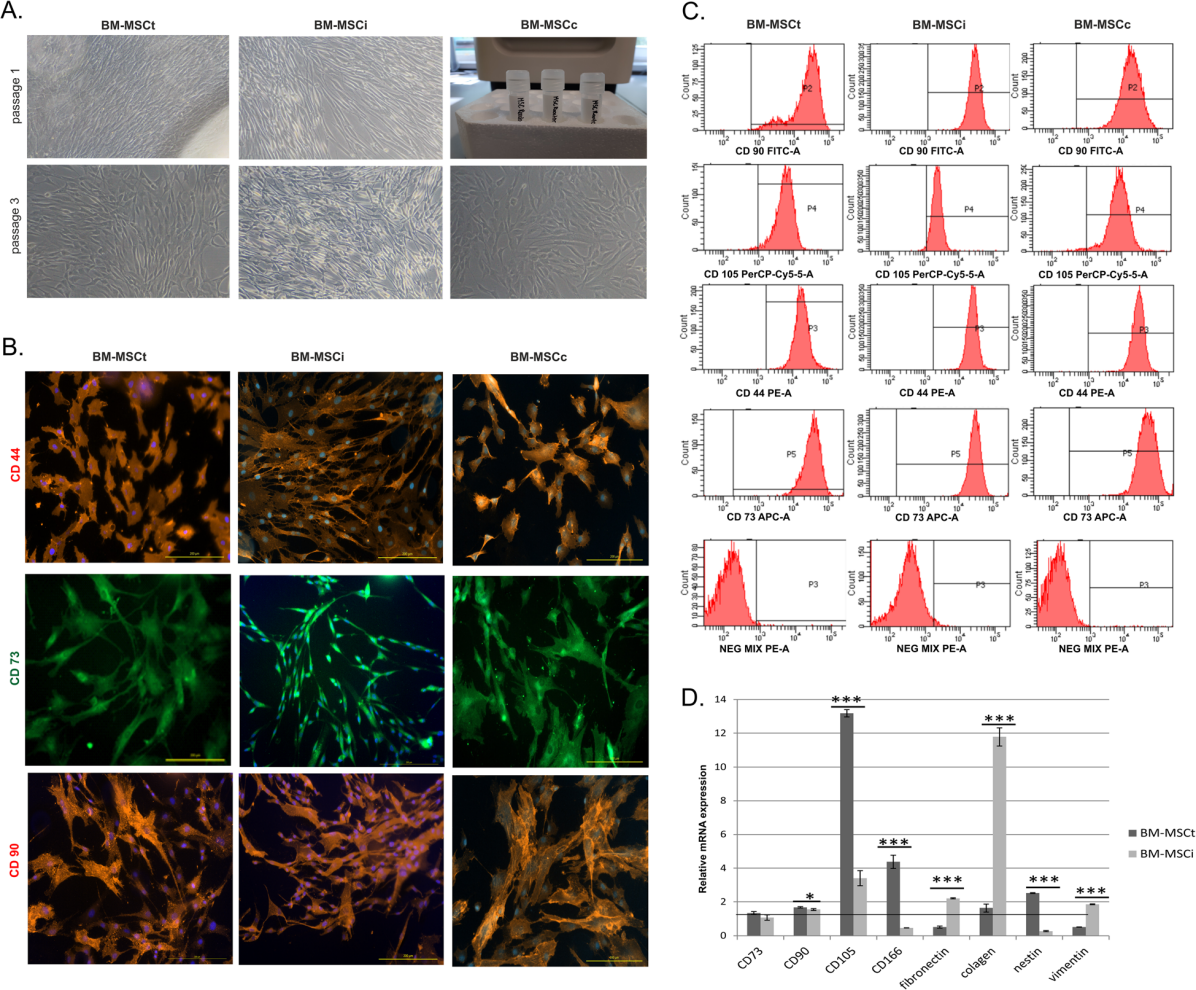
Characteristics of BM-MSC derived from different sources. **a** Morphological image of BM-MSCt, BM-MSCi, and BM-MSCs at first and third passage—cells exhibited typical fibroblast shaped morphology. **b** The expression of cell surface markers presented on BM-MSCi, BM-MSCi, and BM-MSCc using immunocytochemical staining. **c** FACS analysis of BM-MSCi, BM-MSCi, and BM-MSCc in terms of CD90, CD105, CD44, CD73 , and negative marker expression. **d** Gene expression analysis of MSC markers in cells obtained from three different bone marrow sources. Scale bars = 200 μm. All data reflect mean ± SD from ≥ 5 independent experiments; *p < 0.05, ***p < 0.001

Then, we optimized the isolation procedure of bone marrow cells obtained from hip replacement material. The technical method to obtain mesenchymal cells from this source was successful and reproducible in all patients’ bone marrow samples. In this instance, cells derived from the bone marrow of the femoral shaft possess the properties of typical MSC. The morphology of cells isolated from the bone marrow obtained from the iliac crest and femoral shaft was comparable (Fig. 2a). There was no significant difference in the initial cell growth profile among these two sources. Then we focused on the comparison of BM-MSC fractions isolated from the iliac crest and femur in terms of their phenotype and function. To validate our results, we used also commercially available BM-MSC. The phenotype of BM-MSC did not differ significantly among the three groups. All cells presented typical MSC markers as revealed in immunocytochemical staining and flow cytometry analysis (Fig. 2b,c). Irrespectively of the source of bone marrow, isolated MSC had similar phenotype expression of typical MSC markers (CD73, CD90, CD105, CD166). To evaluate the expression level of these markers, quantitative qPCR analysis was performed. The results of our study confirmed the abovementioned observation. BM-MSC obtained from the iliac crest and femur expressed mRNA for mesenchymal markers. Interestingly, MSC derived from the femoral shaft showed higher expression of CD105 and CD166 in comparison to cells isolated from the iliac crest (Fig. 2d). Additionally, gene expression for vimentin, fibronectin, and collagen type I proteins was performed. These makers had higher expression in BM-MSCi than in BM-MSCt. An interesting result was the mRNA expression of Nestin, a specific marker for progenitor cells which was increased in BM-MSCt samples (Fig. 2d).

### Multilineage differentiation potential of isolated BM-MSC

**Fig. 3:**
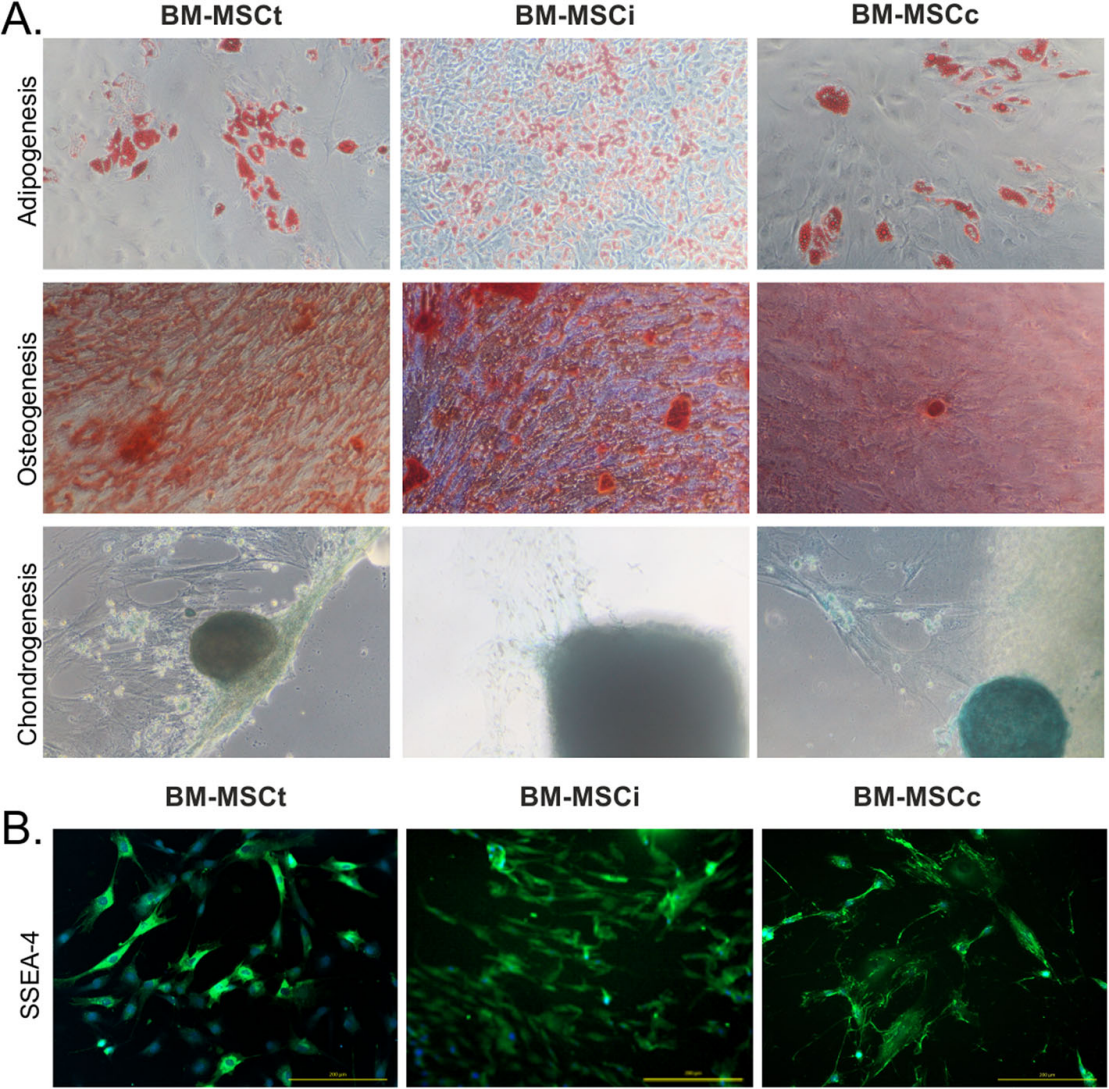
**a** Comparison of multilineage differentiation potential of MSC isolated from the bone marrow of different sources. BM-MSCi, BM-MSCt, and BM-MSCc revealed similar capacity to differentiate into adipocytes (Oil Red staining), osteocytes (calcium deposition inside the cells—stained with Alizarin Red), and chondrocytes (Alcian Blue staining). **b** Expression of SSEA-4 antigen on BM-MSCt, BM-MSCi, and BM-MSCc cells. Scale bars, 200 μm

To analyze cell differentiation potential, three groups of BM-MSC isolated from different sources were induced to undergo multilineage differentiation: adipogenic, chondrogenic, or osteogenic. Adipogenic differentiation of BM-MSC was visible after 7 days of cell culture; the small fat vacuoles were detected. BM-MSCi and BM-MSCt had similar depositions of fat droplets as control BM-MSCc line. Chondrogenic potential of each type of BM-MSC was analyzed in 3-D suspension condition, which naturally mimics cartilage microenvironment. The intensive glycosaminoglycan deposition verified by Alcian Blue staining was observed in all MSCs isolated from the bone marrow of different sources. Similarly, BM-MSCi, BM-MSCt, and BM-MSCc manifested the same potential for osteogenic differentiation. BM-MSC started to present calcium deposits visible by Alizarin Red staining after 10 days of induction (Fig. 3a). Interestingly, in all groups of cells, we observed the expression of stage-specific embryonic antigen-4 (SSEA-4) which is a typical marker for undifferentiated cells; however, it also indicates the multipotential properties of cells [19, 20] (Fig. 3b).

### The growth rate of MSC

**Fig. 4:**
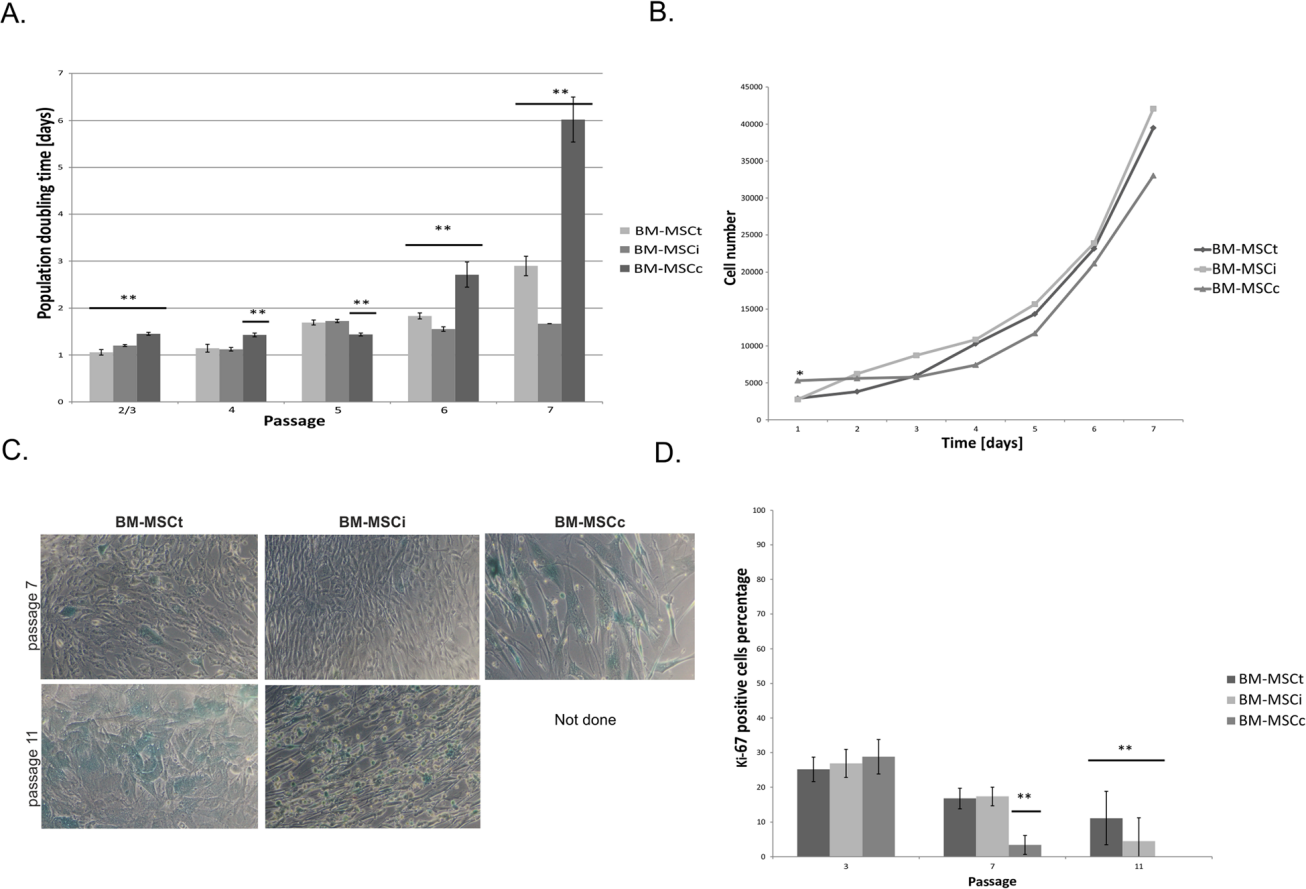
Analysis of BM-MSC cell growth. **a** Population doubling time (PDT, measured in days) determined at each culture of BM-MSCi, BM-MSCt, and BM-MSCc from the 1st to passage 7. **b** The comparison of the growth curves of different BM-MSCs (passage 2/3) calculated based on metabolic activity determined by measuring absorbance at 450 nm wavelength. **c** The representative images of senescence β-galactosidase-positive cells in BM-MSCi, BM-MSCt, and BM-MSCc taken from pictures of five independent experiments. Light blue staining can be observed from passage 7 and gradually increases at passage 11. **d**. Graph representing the percentage of proliferating cells stained with Ki67 antibody. All data reflect mean ± SD from ≥ 5 independent experiments; **p < 0.01

To compare the proliferation rate in bone marrow cells isolated from different sources, long-term analysis based on population doubling time calculation (PDT) was performed. Proliferation analysis demonstrated that the growth curve of BM-MSCt was similar to the growth curve of iliac crest cell BM-MSCi and BM-MSCc. However, BM-MSCt displayed the highest proliferative capacity during a long time in vitro in comparison to cells isolated from two other BM sources (Fig. 4a). The short time analysis based on the metabolic activity of cells measured during 7 days of culture revealed that BM-MSCi and BM-MSCt exhibited similar growth patterns (*p* < 0.01) (Fig. 4b). Next, to examine the number of senescence cells present in BM-MSCi, BM MSCt, and BM-MSCc population, the analysis of β-galactosidase activity was used. Single β-galactosidase-positive cells were detected in all investigated BM-MSCs isolated from three different sources at the stage of passage 7 (Fig. 4c). Then, the increase of positive staining was observed during a longer time of cell culture (11th passage) in BM-MSCi and BM-MSCt. The commercially available BM-MSCc did not proliferate after passage 10; therefore, the experiments using these cells were not performed due to the extremely low cell number (Fig. 4d).

### Paracrine properties/mRNA cytokine secretion analysis

**Fig. 5:**
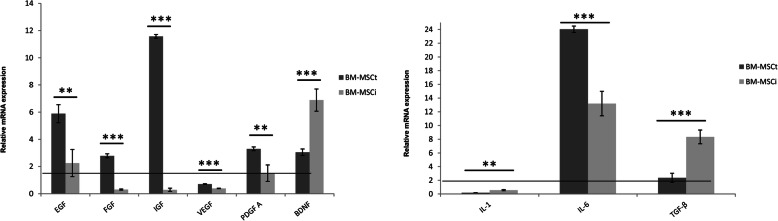
Comparison of gene expression in three types of BM-MSC lines—relative mRNA expression of selective growth factors and cytokines. The relative mRNA expression was calculated using the ΔΔCt method. Gene expression results were normalized to commercial BM-MSCc, using GAPDH as a reference gene. All data reflect mean ± SD from ≥ 5 independent experiments, *p < 0.05; **p < 0.01

To analyze if BM-MSC isolated from the different sources have the same paracrine potential, gene expression of selected growth factors and cytokines which are important for regenerative properties of cells, have been measured quantitatively by RT-PCR. The level of mRNA expression of EGF, FGF, IGF, VEGF, PDGF A, BDNF, IL-1, IL-6, TNFα, and TGFβ was detected in MSC isolated from the bone marrow of iliac crest and femoral shaft (Fig. 5). Interestingly, a much higher mRNA level of EGF, FGF, IGF, VEGF, PDGFA was found in BM-MSCt than BM-MSCi. The analysis of mRNA expression for pro-inflammatory and anti-inflammatory cytokines revealed a low value of gene expression for IL-1 and a high level of mRNA for IL-6 and TGF-β1 in BM-MSCi and BM-MSCt population in comparison to BM-MSCc. The TNFα gene expression was not detected in any of the investigated cell samples.

### BM-MSC derived from femoral shaft cultured in 3D bone scaffolds

**Fig. 6:**
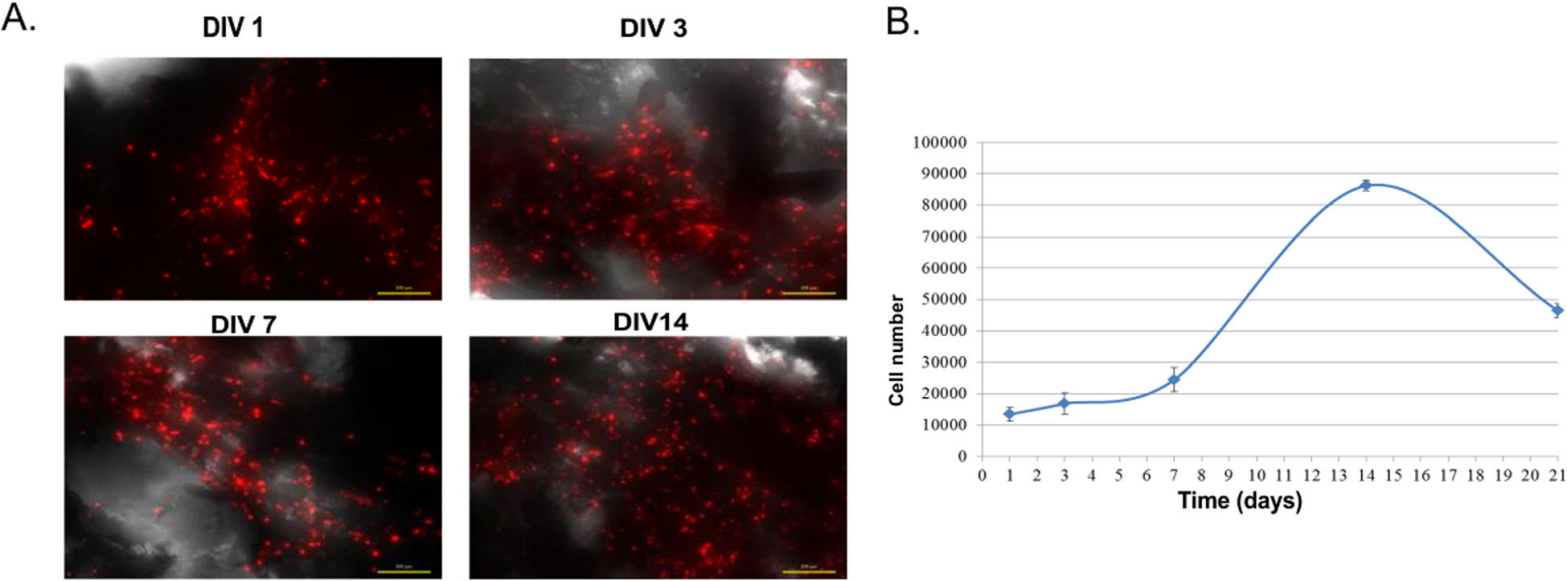
**a** Visual observation of BM-MSCt seeded on 3D bone tissue scaffold stained with Dil (red) during 14 days of culture. **b** Graph presents cell number calculation based on metabolic activity during 21 days of culture. In vitro measurements on days 1, 3, 7, 14, and 21 show increased metabolic activity of BM-MSCt/scaffold till 14 days

The regenerative potential of BM-MSCt has been exploited by co-culture with bone scaffolds that could be used in tissue engineering. Additionally, we tested the interaction between BM-MSCt and cancellous bone tissue scaffold during in vitro co-culture. The main purpose of the study was to trace the behavior and functions of BM-MSCt seeded on bone scaffolds mimicking cell natural microenvironment. The confocal microscope analysis showed that BM-MSCt successfully attached to the surface of scaffolds 24 h after cell seeding and remained there until the end of observation (14 days) (Fig. 6a). We noticed that BM-MSCt effectively incorporated into bone tissue scaffold; moreover, cells maintained viable and proliferated within the scaffold (Fig. 6a). The measurement of BM-MSCt metabolic activity demonstrated that the cell number rose exponentially untill 14 days, then the gentle fall of cell number was observed (Fig. 6b). These preliminary experiments confirmed the cytocompatibility of the scaffold for BM-MSCt attachment. Moreover, we noticed a significant difference in bone tissue structure in the presence or absence of BM-MSCt. 3D bone scaffolds containing BM-MSCt showed the proper tissue architecture during 30 days of in vitro observation and did not degrade in contrast to the group of scaffolds without seeded cells where the process of bone tissue degradation started after 7 days of culture.

## Discussion

Mesenchymal cells are very promising cells for their potential use in regenerative medicine; however, positive results of their transplantation in human cell therapies are not so evident. Still, we need more comprehensive studies confirming the efficacy of these cells to translate experimental results to the clinic [7]. There are a lot of protocols for MSC isolation from various tissues; however, the researchers and clinicians are still facing challenges on how to obtain a pure population of primary MSC with regenerative features [21]. Among different sources of MSC, bone marrow represents the most exploited source. However, the initial number of isolated BM-MSC is still limited [1, 2, 22]. Therefore, to cover the need for a large number of cells for testing, readily available, the appropriate method for MSC isolation needs to be established.

In our preliminary studies, our task was to establish the most optimal procedure for hBM-MSC isolation and further optimize their culture conditions. Briefly, three different methods of BM-MSC isolation from the iliac crest have been performed. Along we tested the gradient separation centrifugation method: standard Ficoll-Paque protocol and 17.5% sucrose procedure which was specifically designed for direct clinical application, appeared easy, safe, and fast (5 min), allowing the collection of a ready-to-use MSC fraction [17]. We have also investigated the effectiveness of the isolation of MSC by using the whole bone marrow seeding technique, the method recently recommended for a large scale of MSC application [22] and compared it with the abovementioned gradient methods. The results of our study have shown that the most effective method for human BM-MSC isolation was the Ficoll-Paque gradient where we obtained a large number of cells in a relatively short time. The characterization of isolated cells has been confirmed following the criteria proposed by the International Society of Cellular Therapy (ISCT). The morphological, phenotypic, proliferative, and differentiation characteristics of BM-MSC were similar in all isolation samples. Based on these findings, we decided to use the Ficoll-Paque protocol to isolate BM-MSC in our further experiments. Most of the current research that focuses on the regenerative potential of human MSC often use bone marrow aspirates obtained from iliac crests of healthy donors. Searching alternatives to the iliac crest as a source of bone marrow seems to be a great challenge that could be addressed.

In this study, we proposed the new source of BM in which the femoral shaft is processed during total hip arthroplasty. We took advantage to use this source of bone marrow to isolate and expand mesenchymal stem/stromal cells (BM-MSCt). We also evaluated the potential of BM-MSCt to differentiate into adipogenic, chondrogenic, or osteogenic lineages since the multipotential capacity has been considered as an important quality of MSC potential [11]. The next step was the determination of the antigens for undifferentiated cells and the senescence-associated phenotype. We have shown that bone marrow obtained from the femoral shaft usually discarded after THA contained a lot of multipotential BM-MSC. According to our observation, BM-MSCt shared common features with BM-MSCi and BM-MSCc, along with the same phenotypic markers and differentiation potential into all mesodermal lines. The growth rate of BM-MSC isolated from different sources revealed that BM-MSCt population presents the most effective proliferation. Additional analysis showed the expression of nestin and SSEA-4, typical markers for undifferentiated cell types. The expression of these markers could be useful as a diagnostic method to distinct proper stemness in the MSC population with regenerative features for subsequent medical applications. The highest expression of nestin was observed in BM-MSCt, which according to the recently published data positively correlated with their multipotent properties [23, 24]. In the present study, we also observed significantly increased mRNA expression of IL-6 in BM-MSCt. The recent experimental data indicated that IL-6 is important in sustaining MSC in an undifferentiated state, and it has a positive impact on proliferation and protection MSC from apoptosis [25]. In our studies, we have also shown the benefit of BM-MSCt as a source of a variety of growth factors. The expression of EGF, FGF, IGF, and PDGF-A at mRNA level was much higher in BM-MSCt than in BM-MSCi. Finally, we also tested the impact of BM-MSCt seeded on bone tissue fragments. Embedded BM-MSCt effectively incorporated into the bone tissue scaffold and started to proliferate. Interestingly, we observed the increased rate of survival of bone tissues as opposed to individual bone fragments without seeded cells. It might provide evidence of the positive effect of MSC-secreted factors into the microenvironment. Based on the above-described information, it is interesting to study whether implantation of BM-MSC could contribute to the repair of bone tissue after transplantation.

In the face of our results, the new source of bone marrow from the femoral shaft that we proposed may prove to be very valuable in terms of research, banking, and transplantation. A comprehensive study of cells obtained from the various bone marrow regions and comparison of different methods of isolation as well as the characteristic of BM-MSC derived from different sources has not been attempted before, and therefore, our findings are new and clinically promising.

## Conclusions

We demonstrated a new alternative source of human bone marrow MSC derived from the femoral shaft processed during total hip arthroplasty. According to our findings, MSC derived from the bone marrow of femoral shaft shares similar biological characteristics with MSC taken from iliac crest bone marrow aspirate. THA-derived bone marrow MSC population contains multipotent stem cells, which have high differentiation capability and can give rise to osteoblasts, chondrocytes, or adipocytes. Moreover, these cells express various growth factors that might be important for tissue regeneration in terms of their transplantation in different disease disorders. The results we have received recently could have a clinical impact in creating a new source of MSC adapted to the future therapy, namely, the isolation and enrichment of BM-MSC during orthopedic surgery and using such cells for replacement therapy either by direct autologous or allogeneic transplantation.

## Data Availability

The datasets used and/or analyzed during the current study are available from the corresponding author on reasonable request.

## Abbreviations

BM-MSC: Bone marrow-derived mesenchymal stem/stromal cells
EGF: Epidermal growth factor
FGF: Fibroblast growth factor
GAPDH: Glyceraldehyde 3-phosphate dehydrogenase
IGF: Insulin-like growth factor 1
IL: Interleukin
MNC: Mononuclear cells
MSC: Mesenchymal stem/stromal cells
PDGF-A: Platelet-derived growth factor subunit A
PDT: Population doubling time
PFA: Paraformaldehyde
PBS: Phosphate-buffered saline
SSEA-4: Stage-specific embryonic antigen-4
THA: Total hip arthroplasty
